# Reply to Lee, S.Y. Comment on “Matsuo et al. Impact of Olfactory Change on Postoperative Body Weight Loss in Patients with Gastric Cancer after Gastrectomy. *Nutrients* 2024, *16*, 851”

**DOI:** 10.3390/nu16152423

**Published:** 2024-07-26

**Authors:** Hiromi Matsuo, Ryota Matsui, Koshi Kumagai, Satoshi Ida, Yoko Saino, Aya Fujiwara, Kumi Takagi, Yukiko Itami, Misuzu Ishii, Naoki Moriya, Yuna Izumi-Mishima, Kazuhiro Nomura, Yasuo M. Tsutsumi, Souya Nunobe, Rie Tsutsumi, Hiroshi Sakaue

**Affiliations:** 1 Department of Nutrition and Metabolism, Institute of Biomedical Sciences, Tokushima University Graduate School, 3-18-15 Kuramoto, Tokushima 770-8503, Japan; h.matsuo.job@gmail.com (H.M.); hsakaue@tokushima-u.ac.jp (H.S.); 2 Department of Nutrition Management, Cancer Institute Hospital of JFCR, 3-8-31 Ariake, Koto-ku, Tokyo 135-0063, Japan; 3 Department of Gastroenterological Surgery, Cancer Institute Hospital of JFCR, 3-8-31 Ariake, Koto-ku, Tokyo 135-0063, Japan; 4 Department of Upper Gastrointestinal Surgery, Kitasato University Hospital, 1-15-1 Kitasato, Minami-ku, Sagamihara-shi 252-0375, Japan; 5 Department of Gastroenterological Surgery, Kumamoto University Hospital, 1-1-1 Honjo, Chuo-ku, Kumamoto-shi 860-8556, Japan; 6 Department of Anesthesiology and Critical Care, Hiroshima University, 1-2-3 Kasumi, Hiroshima 734-8551, Japan

Dr. Sang Yeoup Lee [1] recently provided feedback on our investigation concerning the impact of olfactory change on weight loss post-gastrectomy in gastric cancer patients [2]. Dr. Lee’s correspondence pointed out a discrepancy between our findings and a recent study indicating that pylorus-preserving gastrectomy (PPG) correlates with reduced weight loss and improved maintenance of nutritional status. In response, we re-evaluated our data, focusing on the various surgical procedures performed. We performed Wilcoxon’s rank-sum test for comparing two groups lacking correspondence and calculated the median (interquartile range, IQR) for non-normally distributed continuous variables. The percentage of weight loss observed following each procedure was as follows: total gastrectomy (*n* = 10): 9.3 (8.0–11.7)%, phrenic gastrectomy (*n* = 12): 8.3 (6.7–7.9)%, pyloric gastrectomy (*n* = 27): 4.6 (3.2–7.0)%, and pyloric preservation gastrectomy (*n* = 9): 5.8 (4.9–6.2)%. Consistent with prior studies, patients undergoing total gastrectomy exhibited a higher rate of weight loss [3]. These results are consistent with previous reports, suggesting that our results exhibit no discernible deviation from the expected trends. Subsequently, we investigated the impact of each surgical procedure on olfactory changes by conducting a two-group comparison between patients with and without postoperative olfactory changes. In the total gastrectomy group, the percentage of patients experiencing olfactory changes was 8.1% (7.2–8.5) in the no olfactory change group (*n* = 6) and 12.2% (11.5–12.4) in the olfactory change group (*n* = 4), indicating a significant decrease in the group with olfactory changes (*p* = 0.019). Conversely, in the pyloric preservation gastrectomy group, there was no statistically significant difference in weight loss between patients with 5.8 (5.5–6.9)% and without 6.2 (3.5–6.2)% olfactory changes (*p* = 0.849). Following distal gastrectomy, the no olfactory change group (*n* = 11) experienced a 7.9 (6.5–8.9)% reduction in body weight, while the olfactory change group (*n* = 1) exhibited a 9.1% decrease in body weight. Among patients undergoing pyloric gastrectomy, those without olfactory changes (*n* = 26) detected a 4.6 (3.2–7.0)% decrease in body weight, whereas those with olfactory changes (*n* = 1) lost 9.2% of their body weight. These findings suggest that olfactory dysfunction may significantly contribute to weight loss across different surgical procedures. However, given the relatively small sample size, the high number of patients with olfactory impairment in the PPG group may be attributed to random error.

Dr. Lee further emphasized the link between ghrelin and olfactory changes. In our investigation, we acknowledged the potential similarity in the mechanism of olfactory impairment observed in gastric cancer patients after gastrectomy and that seen in bariatric surgery. We posited that ghrelin, in conjunction with vagal pathways, leptin, and gastrointestinal symptoms, may play a pivotal role in the mechanism underlying olfactory changes post-gastrectomy. As outlined in our paper, bariatric surgery often precipitates an increased incidence of olfactory changes alongside significant initial and long-term weight loss. Ghrelin has been found to bolster functional connections between the hippocampus and ventral striatum, thereby causing individuals to perceive food odors more favorably following intravenous ghrelin administration [4]. Among the olfactory changes in this study, olfactory hypersensitivity occurred more frequently. Olfactory assessments have indicated that individuals with hypersomnia seldom display lowered thresholds and frequently perceive odors as unpleasant. [5]. Gastrectomy led to diminished ghrelin levels, subsequently dampening the signaling of the meal-induced reward system, resulting in food aversion. Additionally, glucagon-like peptide (GLP-1) may also play a role in this process. Research has indicated reduced activation of the brain’s reward center and aversion to sweet/fatty foods in obese patients following bariatric surgery, possibly attributable to elevated GLP-1 levels [6,7]. Similarly, GLP-1 levels are elevated after gastrectomy for gastric cancer, suggesting that gut–brain communication via GLP-1 induction may contribute to the observed olfactory changes.

Finally, Dr. Lee queried our rationale for employing the visual analog scale (VAS) to assess olfactory changes. The Japanese guideline for olfactory changes recommends VAS as a self-assessment tool for quantifying subjective olfactory function [4]. We opted for VAS in this study due to its simplicity, non-invasiveness, and suitability for clinical application. However, recognizing that olfactory hypersensitivity may be influenced by psychological factors such as unpleasant emotions, we acknowledge the necessity for more objective and quantitatively robust testing methods. This limitation is duly noted in our paper. Moving forward, it is imperative to evaluate olfactory changes in a more objective and quantitative fashion and ascertain whether objective olfactory assessment correlates with postoperative weight loss.

In our study, we have proposed the potential significance of olfactory changes following gastrectomy in gastric cancer patients as an independent predictor of weight loss. However, further exploration is warranted to elucidate the intricate underlying mechanisms. As highlighted by Dr. Lee, a comprehensive investigation into the mechanisms driving this phenomenon, particularly within the context of the gut–brain axis, is crucial. Such inquiry may unveil these mechanisms as the primary drivers of weight loss, with olfactory changes serving as a mediating factor. Nonetheless, given the ease and non-invasiveness of evaluating olfactory changes, integrating them as a nutritional indicator holds promise for enhancing dietary intake and satisfaction.

## Abbreviations

Pylorus-preserving gastrectomy (PPG), visual analog scale (VAS), interquartile range (IQR).
